# Supplementary material for: The Effect of Drying Methods and Extraction Techniques on Oleuropein Content in Olive Leaves
Source: Plants (Basel). 2022 Mar 24;11(7):865. doi: 10.3390/plants11070865 (PMC9003305; doi:10.3390/plants11070865)
Supplement: Supplementary file 1 [file plants-11-00865-s001.zip › plants-1607968-supplementary.pdf]

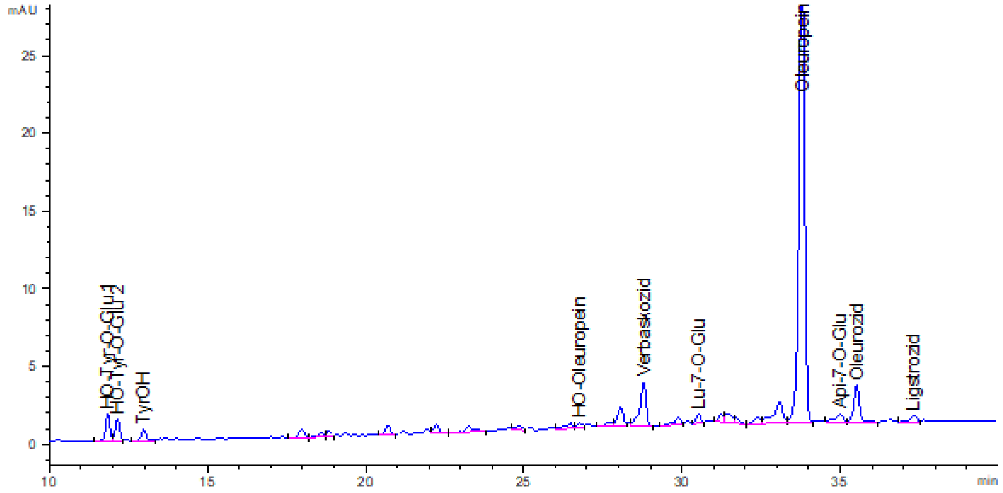

Figure S1: Chromatogram of Istrska belica air drying (room T) – DES1

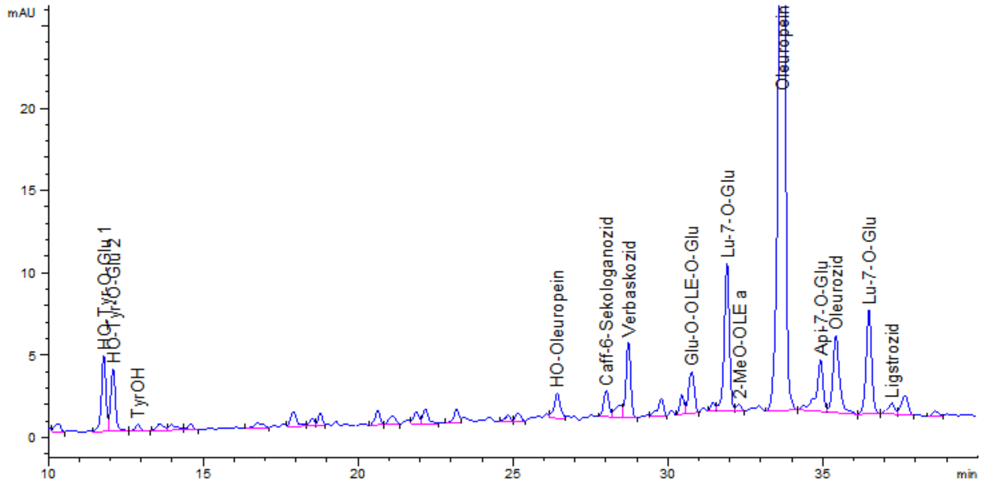

Figure S2: Chromatogram of Istrska belica – air drying (T=105°C) – DES2

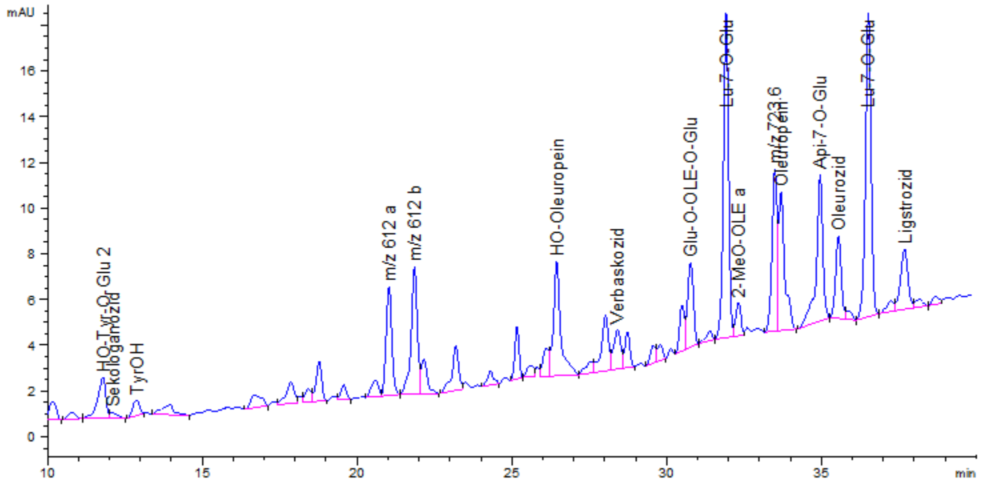

Figure S3: Chromatogram of Istrska belica - freeze drying – DES3

Table S1. Content of active compound (C) in mg_c/ g d.w. of active compounds of *Istrska belica* and *Leccino* methanol extracts at a different type of drying. Data are means ± SD from three replicates.

Type	*Istrska belica* - air drying (room T)	*Istrska belica* - air drying (T=105°C)	*Istrska belica* - freeze drying	*Leccino* - air drying (room T)	*Leccino* - air drying (T=105°C)	*Leccino* - freeze drying
**Compound**	**mg c / g d.w.**					
**2-MeO-OLE a[1]**	n.d.	n.d.	2.71±0.06	n.d.	n.d.	1.21±0.02
**2-MeO-OLE b[2]**	n.d.	n.d.	2.22±0.05	n.d.	n.d.	1.10±0.02
**Api[3]**	1.5±0.03	1.11±0.02	0.51±0.01	1.71±0.04	1.31±0,03	0.70±0.01
**HO-Ole[4]**	1.01±0.02	1.40±0.03	1.63±0.03	1.11±0.02	1.10±0,02	1.10±0.02
**HO-Tyr-O-Glu1[5]**	3.21±0.07	4.31±0.09	0.72±0.01	6.73±0.14	9.01±0,19	0.81±0.02
**HO-Tyr-O-Glu2[6]**	2.60±0.05	3.12±0.07	0	6.91±0.14	6.32±0,13	n.d.
**Lig[7]**	1.21±0.03	1.10±0.02	0.91±0.02	1.02±0.02	0.71±0,01	0.40±0.01
**Lu-7-O-Glu[8]**	0.92±0.02	1.51±0.03	2.12±0.04	2.62±0.05	1.32±0,03	1.30±0.03
**m/z 723.6[9]**	n.d.	n.d.	2.24±0.05	n.d.	n.d.	3.11±0.07
**Ole[10]**	77.71±1.63	70.10±1.47	4.93±0.11	66.10±1.39	60.51±1,27	3.32±0.07
**Ols[11]**	6.10±0.13	4.40±0.09	0.92±0.02	7.71±0.16	6.50±0,14	1.01±0.02
**Sec[12]**	n.d.	n.d.	1.31±0.03	n.d.	n.d.	1.50±0.03
**TyrOH[13]**	1.52±0.03	0.70±0.01	0.53±0.01	2.82±0.06	1.08±0,02	0.80±0.02
**Ver[14]**	6.90±0.14	1.40±0.03	0.61±0.01	6.13±0.13	1.61±0,03	0.81±0.02

[1]Methoxy-oleuropein a, [2]Methoxy-oleuropein b, [3]Apigenin, [4]Hydroxyoleuropein, [5]Hydroxytyrosol glucoside 1, [6]Hydroxytyrosol glucoside 2, [7]Ligstroside, [8]Luteolin-7-Glucoside, [9]isomer m/z 723.6, , [10]Oleuropein, [11]Oleuroside, [12]Secologanoside, [13] Hydroxytyrosole, [14]Verbascoside; [16]not defined; SD ± means the standard deviation

Table S2: Content of active compound (C) in mg_c / g d.w. in eutectic extracts of *Istrska belica* and *Leccino*. Data are means ± SD from three replicates

Type	DES1	DES2	DES3	DES4	DES5	DES6
**Compound**			**mg_c / g d.w.**			
**Api-7-O-Glu[1]**	1.91 ±0.03	2.61 ±0.05	1.12 ±0.02	3.1 ±0.06	3.92 ±0.07	1.41 ±0.02
**Glu-O-OLE-O-Glu[2]**	1.72 ±0.03	1.71 ±0.03	0.63 ±0.01	1.8 ±0.03	2.31 ±0.04	0.3 ±0.01
**HO-Ole[3]**	1.21 ±0.02	1.12 ±0.02	1.2 ±0.02	0.9 ±0.02	1.24 ±0.02	0.41 ±0.01
**HO-Tyr-O-Glu 1[4]**	1.91 ±0.03	2.51 ±0.05	n.d.	3.81 ±0.07	5.91 ±0.11	n.d.
**HO-Tyr-O-Glu 2[5]**	1.61 ±0.03	2.11 ±0.04	0.41 ±0.01	4.22 ±0.08	5.51 ±0.09	0.40 ±0.01
**Lig[6]**	0.71 ±0.01	0.52 ±0.01	0.51 ±0.01	1.11 ±0.02	0.53 ±0.01	0.32 ±0.01
**Lu[7]**	n.d.[18]	n.d.	n.d.	0.22 ±0.01	0.23 ±0.01	n.d.
**Lu-7-O-Glu[8]**	3.41 ±0.06	4.13 ±0.08	2.2 ±0.04	2.53 ±0.05	3.11 ±0.06	2.90 ±0.5
**m/z 612 a[9]**	n.d.	n.d.	0.73 ±0.01	n.d.	n.d.	0.21 ±0.01
**m/z 612 b[10]**	n.d.	n.d.	0.92 ±0.01	n.d.	n.d.	0.41 ±0.011
**m/z 723.6[11]**	n.d.	n.d.	1.1 ±0.02	n.d.	n.d.	1.21 ±0.02
**Ole[12]**	45 ±0.81	43.12 ±0.86	0.92 ±0.02	30.22 ±0.54	43.41 ±0.78	0.30 ±0.01
**Ols[13]**	3.72 ±0.07	3.73 ±0.07	0.52 ±0.01	3.91 ±0.07	5.12 ±0.09	0.4 ±0.01
**Sec[14]**	n.d.	n.d.	0.13 ±0.01	n.d.	n.d.	n.d.
**TyrOH[15]**	1.03 ±0.01	0.22 ±0.01	0.12 ±0.01	1.61 ±0.03	0.35 ±0.01	0.31 ±0,01
**Ver[16]**	7.31 ±0.13	2.51 ±0.05	0.32 ±0.01	6.6 ±0.12	2.21 ±0.04	0.21 ±0.01
**Caff-6-sec[17]**	1.30 ±0.02	0.92 ±0.02	n.d.	0.61 ±0.01	0.61 ±0.01	n.d.

[1]Apigenin-7-glucoside, [2]Glucoside-oleuropein, [3]Hydroxyoleuropein, [4]Hydroxytyrosol glucoside 1, [5]Hydroxytyrosol glucoside 2, [6]Ligstroside, [7]Luteolin, [8]Luteolin-7-Glucoside, [9]isomer m/z 612 a, [10]isomer m/z 612 b, [11]isomer m/z 723.6, [12]Oleuroside, [13]Oleuropein, [14]Secologanoside, [15]Hydroxytyrosole, [16]Verbascoside, [17]Caffeoyl-6-secologanoside; [18]Istrska belica - air drying (room T), [19]Istrska belica - air drying (T=105°C), [20]Istrska belica - freeze drying, [21]Leccino - air drying (room T), [22]Leccino - air drying (T=105°C), [23]Leccino - freeze drying; [18]not defined; SD ± means the standard deviation
